# Analysis of lettuce transcriptome reveals the mechanism of different light/dark cycle in promoting the growth and quality

**DOI:** 10.3389/fpls.2024.1394434

**Published:** 2024-07-09

**Authors:** Mengdi Dai, Xiangfeng Tan, Ziran Ye, Xuting Chen, Yi Zhang, Yunjie Ruan, Bin Ma, Dedong Kong

**Affiliations:** ^1^ Institute of Digital Agriculture, Zhejiang Academy of Agricultural Sciences, Hangzhou, China; ^2^ Hangzhou Institute for Advanced Study, University of Chinese Academy of Sciences (UCAS), Hangzhou, China; ^3^ lnstitute of Agricultural Bio-Environmental Engineering, College of Bio-systems Engineering and Food Science, Zhejiang University, Hangzhou, China; ^4^ Academy of Rural Development, Zhejiang University, Hangzhou, China; ^5^ Institute of Soil and Water Resources and Environmental Science, College of Environmental and Resource Sciences, Zhejiang University, Hangzhou, China; ^6^ Hangzhou Global Scientific and Technological Innovation Center, Zhejiang University, Hangzhou, China

**Keywords:** *Lactuca sativa*, transcriptome, L/D cycle, growth, quality

## Abstract

Light/dark (L/D) cycle plays a crucial role in controlling the production and quality of vegetables. However, the mechanism of L/D cycle on vegetable growth and quality is scarce studied. To investigate the impact of L/D cycle on lettuce growth and quality, we designed three diel scenarios, including 16 hours of light and 8 hours of darkness (L16/D8), 12 hours of light and 6 hours of darkness (L12/D6), and 8 hours of light and 4 hours of darkness (L8/D4). By phenotypic analysis, we found that lettuce grew taller under the L8/D4 scenario than under L16/D8 light cycle scenarios. The physiological indexes showed that the lettuce leaves grown in the L8/D4 scenario exhibited greater enhancements in the levels of soluble protein, soluble sugar, and carotenoid content compared to the other scenarios. By comparing the expression levels under different diel scenarios (L16/D8 vs L12/D6, L16/D8 vs L8/D4, and L12/D6 vs L8/D4), we identified 7,209 differentially expressed genes (DEGs). Additionally, 3 gene modules that were closely related to L/D cycle of lettuce were selected by WGCNA analysis. The eigengenes of three gene modules were enriched in plant hormone signal transduction, sphingolipid metabolism, and nucleocytoplasmic transport pathways. Through network analysis, we identified six hub genes (CIP1, SCL34, ROPGEF1, ACD6, CcmB, and Rps4) in the three gene modules, which were dominant in plant circadian rhythms and greatly affected lettuce growth. qRT-PCR analysis confirmed the diurnal response patterns of the 6 hub genes in different treatments were significant. This study intensively enhanced our comprehension of the L/D cycle in the growth morphology, nutritional quality, and metabolic pathways of lettuce.

## 1 Introduction

Vegetables, as high-value-added crops, are crucial for human well-being. Despite vegetables accounting for a minor proportion of overall crop production in comparison with cereals, their significant fourfold yield makes these crops of major focus (FAO online). Nevertheless, the cultivation of vegetables is still greatly hindered by inadequate water and mineral nutrient resources, a decrease in available workforce, and diminished soil fertility (Nkebiwe et al., 2016; Wang et al., 2016; Zhang and Guo, 2016). The development of plant factory has made a swift progress in the production of vegetable food. This progress is anticipated to address issues such as shortage or instability of vegetable supply, resource efficiency, and the environmental impact of agricultural activities (Kozai and Niu, 2016; Kozai, 2018). Lettuce (*Lactuca sativa* L.) is a main vegetable crop cultivated in plant factories. It is widely consumed because of its good taste and high nutritional value (Bian et al., 2018). Lettuce has been extensively studied as a model plant to investigate the impact of light intensity, quality, and duration on plant phenotype and physiology.

In ecosystem, the most prominent cyclic environmental cue of the day is the light/dark cycle, which directly drives the temperature cycle and influences the foraging of animals and the growth of plants. Light/dark (L/D) cycles are characterized by the length of cycle (period) and the ratio of illumination to darkness (L/D ratio). When the L/D cycle period equals the diurnal cycle period 24 h, it is considered normal; otherwise, it is considered abnormal (Bowsher et al., 1991; Cheung et al., 2014). The use of unusual or non-standard light/dark cycles is permitted in closed plant production systems since there is no externally imposed 24-hour cycle (Chen et al., 2022). It has been suggested that the abnormal L/D cycle generated by artificial light might enhance the production of valuable secondary metabolites by plant cells (Zhang et al., 2021). In cultivated wheat (*Triticum aestivum*), compared with a 12 h cycle, the 6 hour L/D cycle facilitated growth and development and resulted in faster ear emergence, further suggesting that 12 h darkness was excessive (Clauw et al., 2024). On solanaceae plants, plant growth, development, photosynthetic pigment concentration, anthocyanin and flavonoid content, and redox state were all impacted by extended light/dark cycles of 24/12 h, 48/24 h, 96/48 h, 120/60 h, and 360/0 h (Shibaeva et al., 2024). Long light plants (lettuce and basil, respectively) cultivated indoors could perform and yield better when exposed to a decreased photoperiod with intermittent modes of light (Avgoustaki et al., 2021; Chen, 2018). Therefore, finding plants’ appropriate light/dark cycle has a more economic, sustainable, commercial and ecological impact on the energy supply for indoor food production. The circadian clock serves as a timing mechanism within plants that enables organisms to adapt to physiological and behavioral changes in the cyclical environment, such as the L/D cycle or food availability. When the external L/D cycles were frequently reversed, the circadian rhythmic gene expression was disturbed (Yang et al., 2024). In Arabidopsis, circadian genes changed the oscillation cycle to match the total duration of the light-dark cycle, resulting in more photosynthesis and better survival (Yari Kamrani et al., 2022). In tomato, circadian genes *EID1* and *LNK2* had positive response to different light periods (Xiang et al., 2022). However, few studies have investigated the effects of L/D cycle on the gene expression in lettuce, especially from a perspective of transcriptome.

This study aimed to explore the effects of different light/dark cycles on the phenotype and quality of lettuce by designing different diel scenarios. Through RNA sequencing, hub genes responded to light/dark cycle were excavated These studies contributed to understand the response mechanism of L/D cycle to plant growth, and provide a basis for the development and application of LED light sources in plant factories.

## 2 Materials and methods

### 2.1 Plant materials and growth conditions

From 2 October 2022 to 28 June 2023, experiments were conducted at Zhejiang Academy of Agriculture Sciences (Latitude: 30.30°N, Longitude: 120.19°E), Hangzhou, China, within an artificial climate chamber. Roman lettuce seeds (*Lactuca sativa* L., “Ideal-205”; Ideal Agriculture Technology, Nanjing, China) were planted in a small flowerpot (7 cm diameter and 5 cm height) containing substrate soil (substrate: vermiculite: perlite = 3:1:1), and positioned inside an artificial climate chamber. The climate chamber measured 1,130 mm (L) x 795 mm (W) x 1920 mm (H), consisting of three microclimate chambers and a control system that regulated temperature, relative humidity and LED lighting. The growth conditions were set as a photosynthetic photon flux density (PPFD) of 204.41 ± 4.97 μmol m^−2^ s^−1^ at the upper part of the canopy, while maintaining an air temperature of 25 ± 2°C and a relative humidity of 85%.

**Figure 1:**
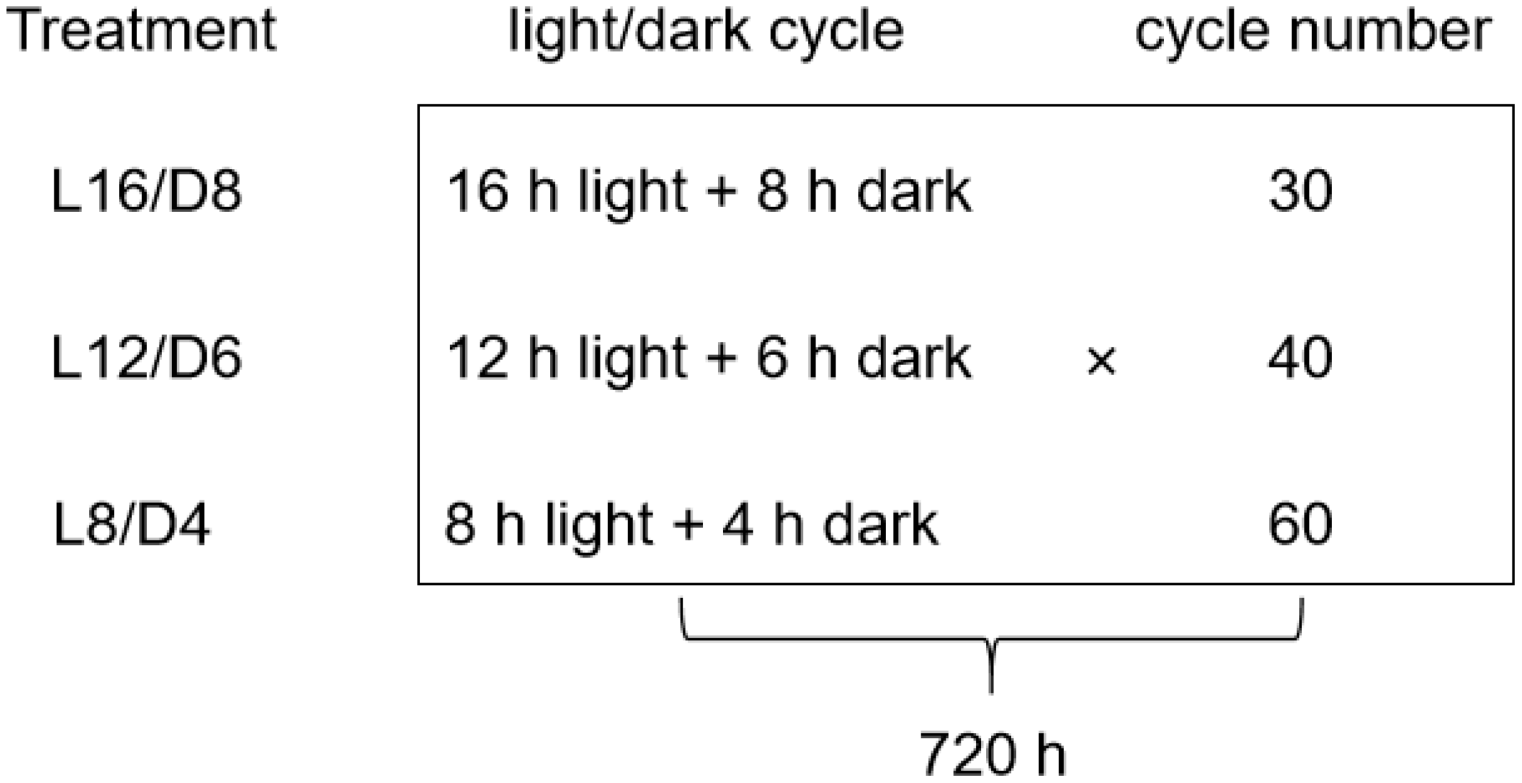
The different L/D cycle in a 720-h growth period. L16/D8, lettuces experience 16 hours of light and 8 hours of darkness a cycle; L12/D6, lettuces experience 12 hours of light and 6 hours of darkness a cycle; L8/D4, lettuces experience 8 hours of light and 4 hours of darkness a cycle. There were totally 30, 40, and 60 L/D cycles in the tested period.

After the second true leaf had fully unfolded, each seedling was transplanted separately into a small flowerpot with a diameter of 7 cm. The pot contains substrate soil as described above. The seedlings were then exposed to three diel scenarios. The three diel scenarios were as follows: (1) 16 hours of light and 8 hours of darkness (L16/D8); (2) 12 hours of light and 6 hours of darkness (L12/D6); (3) 8 hours of light and 4 hours of darkness (L8/D4). The light intensity for three scenarios was set to 207.88 ± 4.92 μmol m^−2^ s^−1^. The LED light devices were bought from Kesheng Experimental Instrument Co., LTD in Ningbo, China. The spectral radiometer (PLA-30, Everfine Optoelectronic Information Co., LTD., Hangzhou, China) was utilized to measure the photon flux (**Supplementary Figure S1**). Each scenario was tested three times. As shown in **Figure 1**, plants were exposed to three different light treatments for 30 days (720 hours). The control was 16 h light/8 h dark (L16/D8), which resulted in totally 30 L/D cycles in the tested period (720 h). Similarly, 12 h light/6 h dark (L12/D6) and 8 h light/4 h dark (L8/D4), which had totally 40 and 60 L/D cycles in the tested period. Following a 30-day period of transplantation, lettuce plants were sampled at a fixed initial planting time point to measure the corresponding parameters. Additional samples were promptly placed in liquid nitrogen and stored at a temperature of -80°C.

### 2.2 Phenotype measurement

After 30 days transplanting, phenotype measurements were conducted within 30 minutes of the lighting switch on. For each scenario, 18 plants randomly selected were regarded as a repetition, and there were 3 repetition in each treatment. The plant height was the linear distance between the highest point of the canopy leaf and the substrate soil, and the maximum canopy diameter was the maximum linear distance between the canopy leaf tip. The leaf was photographed with a Nikon camera, and its area was calculated using the image segmentation approach. The root shoot ratio was computed as follows:


$$\text{Root shoot ratio}=\frac{\text{fresh weight of underground part}}{\text{fresh weight of above}-\text{ground part}}$$

### 2.3 Determination of physiological indexes

For analysis, plant leaves that were in good health and fully developed were selected randomly from each scenario. The determinations were performed within 30 minutes of the lighting switch on. The analysis was repeated 3 times for each physiological index.

#### 2.3.1 Measurement of soluble sugar content

According to the method of Tang (Tang et al., 2018), 0.3 g fresh plant leaves were used to remove the outer layer and subsequently pulverized into powder using liquid nitrogen. The powder was placed into a centrifuge tube with a volume of 15 mL and mixed with 10 mL of ddH_2_O. The mixture was then heated in a boiling water bath for 30 min to extract the liquid. Afterward, the resulting liquid was transferred to a volumeter bottle with a capacity of 25 mL. The measurement of absorbance was conducted at a wavelength of 630 nm, and the content of soluble sugar was determined based on the established standard curve.

#### 2.3.2 Measurement of soluble protein content

The Coomassie bright Blue G-250 method, as described by Bradford (1976), was used to determine the soluble protein content. 1.0 g fresh plant leaves were pulverized using liquid nitrogen, then mixed with 2 mL of ddH_2_O. After being left at room temperature (20-25°C) for 0.5-1 h, the supernatant was collected. 0.6 mL of extraction was added, followed by 5 mL of Coomassie bright blue solution. The mixture was then stirred and left for 2 min. Subsequently, the absorbance was measured at a wavelength of 595 nm, and the content of soluble protein was determined using the standard curve.

#### 2.3.3 Measurement of free amino acid content

0.5 g plant sample were pulverized using liquid nitrogen and then mixed with 5 mL of 10% acetic acid. After that, the mixture was centrifuged and 2 mL of the supernatant was collected. The supernatant was added into acetate buffer (pH=5.4) to 25 mL. Place 2 mL of the sample extract into a test tube with a capacity of 15 mL, then boil it in a water bath for a duration of 15 minutes. The sample dilution was supplemented with 3mL of ninhydrin solution, and the absorbance was measured at a wavelength of 580 nm. The free amino acid content was then determined using the standard curve.

#### 2.3.4 Measurement of chlorophyll content and carotenoid content

According to the method of Xu et al. (2020), 1.0 g fresh plant leaves were pulverized using liquid nitrogen and then mixed with 15 mL of 95% ethanol. Filter the grinding liquid into a 25 mL brown bottle. Transfer the carotenoids from the filter paper to the bottle using a small quantity of 95% ethanol. Next, pour 25 mL of ethanol into the bottle and shake well. The solution for extraction was poured into a colorimetric dish with an optical diameter of 1 cm, using 95% ethanol as the blank. The measurement of absorbance was conducted at the wavelengths of 665 nm, 649 nm, and 470 nm.

#### 2.3.5 Measurement of nitrate

According to the method Cataldo et al. (1975), 2.0 g fresh plant leaves were ground into powder in liquid nitrogen, and added into 20 mL water, 1 mL ammonia buffer, shake 30 min. The homogenate was transferred into 50 mL volumetric bottle, added 0.4 mL of 150 g/L potassium ferrocyanide solution, then added 0.4 mL of 300 g/L zinc sulfate solution. The measurement of absorbance was conducted at a wavelength of 219 nm based on the established standard curve.

### 2.4 RNA sequencing

The same part of mature leaves of lettuce in different light cycle were collected for analysis. For each scenario, three biological samples were chosen and designated as L16/D8_1, L16/D8_2, L16/D8_3, L12/D6_1, L12/D6_2, L12/D6_3, L8/D4_1, L8/D4_2, and L8/D4_3, correspondingly. All samples were harvested within 30 minutes of the lighting switch on and stored in -80°C. TRIzol reagent (Thermo Fisher, Waltham, USA) was used to extract the entire RNA according to the manufacturer’s protocol. Nanodrop2000 (Thermo Fisher, Waltham, USA) was used to determine the quality and strength of the extracted RNA. A total of nine RNA samples were sent to Hangzhou Zhijun Technology Co., Ltd. for library preparation and RNA sequencing. RNA sequencing was performed using the illumina novaseq 6000. Sequencing data quality control involves analyzing statistics of the sequencing data, raw data and quality control data. Trimmomatic (version 0.39) (Bolger et al., 2014) software was used to filter the pair-end reads. Bwa (version 0.7.17) software is used to align the filtered FASTQ sequence to the reference genome of lettuce downloaded from NCBI (https://www.ncbi.nlm.nih.gov/genome/?term=Lactuca+sativa), and output the transcription.

### 2.5 Differential expression analysis and gene annotation

CoverM (version 0.6.1) (https://github.com/wwood/CoverM) software was employed to filter transcription. Using the reads_per_base and method counts functions, the fragments per kilobase of transcript sequence per millions base pairs (FPKM) and feature count were calculated from the filtered transcripts. Gene function and pathway were screened based on Gene Ontology (GO) and Kyoto Encyclopedia of Genes and Genomes (KEGG) database (Kanehisa and Goto, 2000). By input feature count, the analysis of differentially expressed genes (DEGs) was carried out using DESeq2 (Love et al., 2014). The differential gene expression volcano plots and correlation coefficient heatmap for comparison were analyzed using ggplot2 package and corrplot package in R, respectively. The gene ontology (GO) annotations were assigned to each gene by GOseq package (Young et al., 2010). The software KO-Based Annotation system was used to analyze the statistical enrichment of DEGs in the KEGG pathway.

### 2.6 Analysis of gene co-expression network

For the detection of co-expression network and module, the R package (version 3.6) WGCNA (Weighted gene co-expression network analysis) was employed (Langfelder and Horvath, 2008). Pearson’s correlations were conducted to detected the correlations of the overall gene expression levels between biological replicates in the RNA-seq assay (R^2^>0.8). Clusters and heatmaps from the RNA-seq data under different light cycle were analyzed using ggplot2 package according to the FPKM value. The Cytoscape-V3.7.2 and its plugin cytohubba were used to construct the network for the modules. The top 30 core genes in connectivity were visually analyzed.

### 2.7 Verification of gene expression by qRT-PCR assays

**Figure 2:**
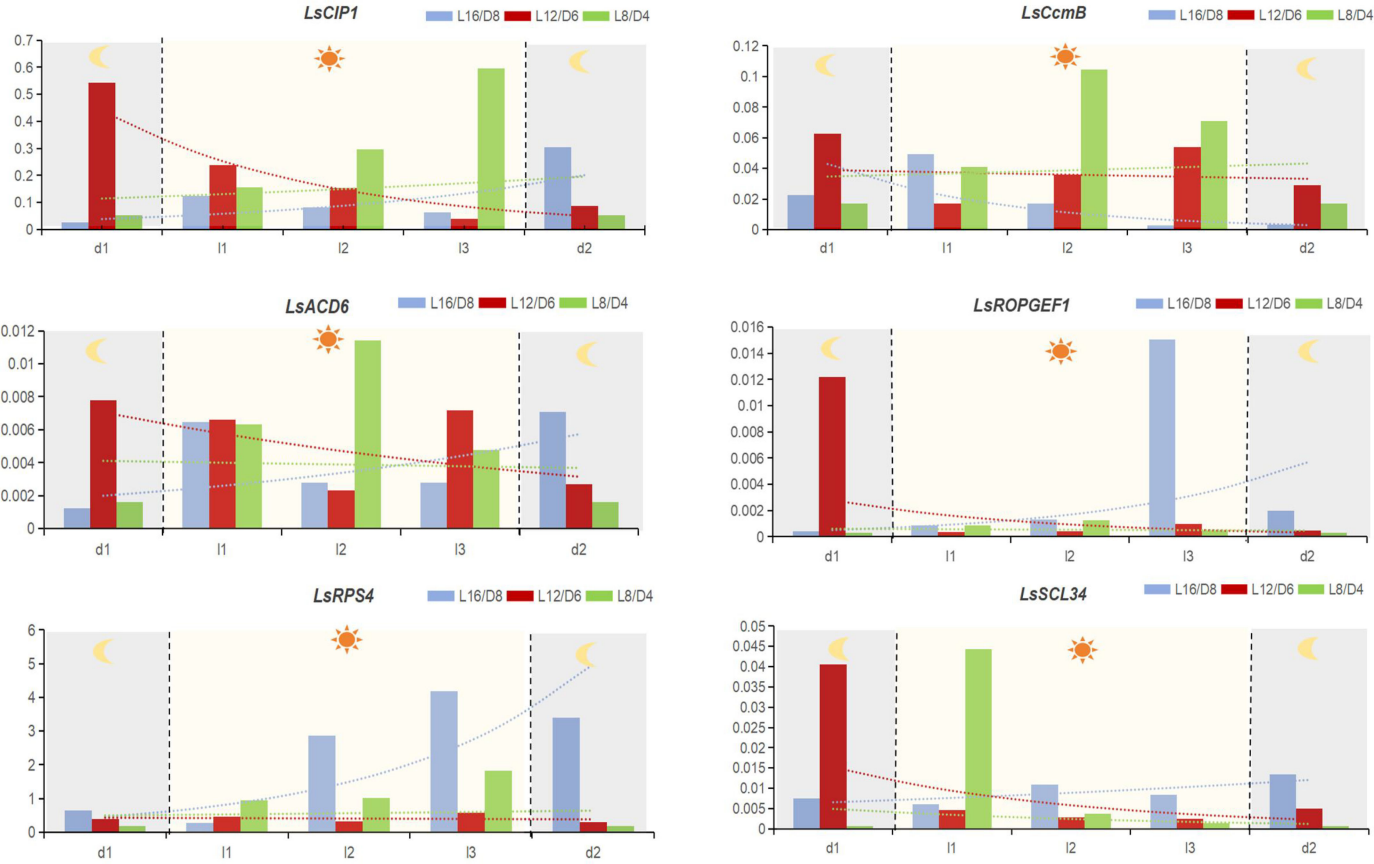
The relative expression level of hub genes in different scenarios. The gene’s relative expression is determined using the 2^-ΔCt^ method. d1, 2 hours before entering the light period; l1, when the lettuces entering the light period. l2, 2 hours after entering the light period; l3, 4 hours after entering the light period; d2, 2 hours after entering the dark period. The data signifies averages and the error bars indicate the standard deviations obtained from six samples that are biological replicates.

The plants in each scenario will return to the same time point to enter the light cycle after going through the 72 h cycle (**Figure 2**). So we sampled the lettuce 2 hours before entering this light period, which is d1; When the lettuces entering this light period, we sampled the materials and remarked as l1; When the lettuces entering this light period 2 hours later, we sampled the materials and remarked as l2; And 4 hours later remarked as l3. Finally, When the lettuces of each scenario entering the subsequent dark phase 2 hours later, we sampled the materials and remarked as d2. 1.0 g fresh plant leaves of different phases were ground into powder in liquid nitrogen. The first-strand cDNA was generated by reverse transcribing total RNA extracted with TRIzol reagent and transcriptase (Takara, Kyoto, Japan). In qRT-PCR, the gene *β-tubulin* (LOC111880334) was used as a control. **Supplementary Table S2** showed a list of the primers used in this study. The 2^-ΔCt^ method was used to determine the relative gene expression (Livak and Schmittgen, 2001; Thomas et al., 2008). Each scenario had three replicates, and the experiments were conducted at least twice.

### 2.8 Statistical analysis

For phenotypic and physiological data, statistical analysis were conducted by one-way analysis of variance (ANOVA), using SPSS 24.0 (SPSS, Inc., Chicago, USA). 18 samples and 3 samples were collected as one repeat for phenotypic and physiological measurement, respectively, and each treatment was repeated three times. The values represented the mean ± standard error. Tukey’s multiple range test was used to determine the differences between mean values at the 0.05 level.

## 3 Results

### 3.1 Effects of different L/D cycle on phenotype of lettuce

**Figure 3:**
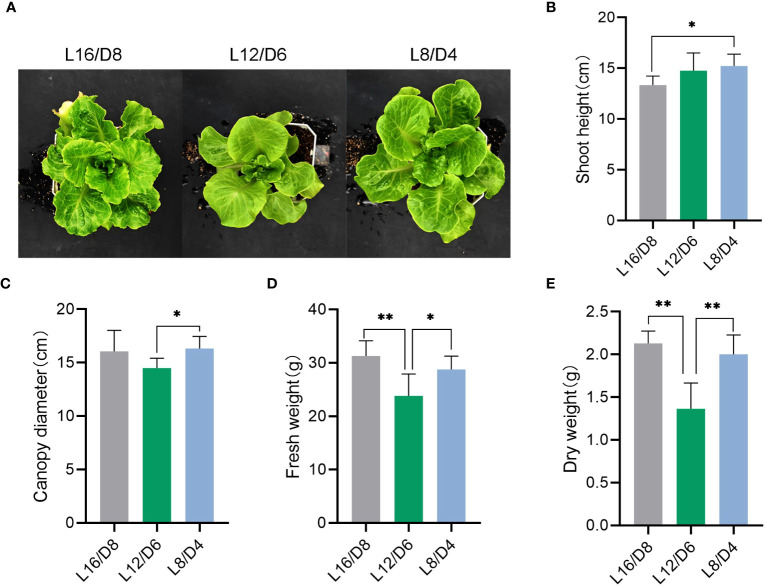
Phenotypic analysis of lettuce under different light cycles. **(A)** The phenotypic image of lettuce growing at L16/D8, L12/D6, and L8/D4. **(B)** The shoot height of lettuce growing at L16/D8, L12/D6, and L8/D4. **(C)** The canopy diameter of lettuce growing at L16/D8, L12/D6, and L8/D4. **(D)** The fresh weight of lettuce growing at L16/D8, L12/D6, and L8/D4. **(E)** The dry weight of lettuce growing at L16/D8, L12/D6, and L8/D4. L16/D8, lettuces experience 16 hours of light and 8 hours of darkness a cycle; L12/D6, lettuces experience 12 hours of light and 6 hours of darkness a cycle; L8/D4, lettuces experience 8 hours of light and 4 hours of darkness a cycle. The data signifies averages and the error bars indicate the standard deviations obtained from six samples that are biological replicates. *P<0.05, **P<0.01.

To investigate the sensitivity of lettuce to different L/D cycle, we measured plant shoot, canopy diameter and fresh and dry weight of lettuce grown at L16/D8, L12/D6 and L8/D4. We found that the phenotype of lettuce changed significantly under different L/D cycle (**Figure 3A**). Lettuce that experienced diel change twice a day (L8/D4) were significantly taller than lettuce that experienced diel change only once (L16/D8) (**Figure 3B**), indicating that different L/D cycle could affect lettuce growth (*p*<0.05). Canopy diameter plays a crucial role in plant growth and fruit bearing. From the results, it was found that the diameter of the canopy of lettuce in L8/D4 scenario was significantly larger than that of lettuce in L12/D6 scenario (**Figure 3C**) (*p*<0.05). In addition, in terms of fresh weight and dry weight, lettuce in L8/D4 scenario was not much different from lettuce in L16/D8 scenario, but both were heavier than lettuce in L12/D6 scenario (**Figures 3D, E**) (*p*<0.05). By measuring the root shoot ratio and leaf area, it was found that the underground part of lettuce growing under L16/D18 was more developed (**Supplementary Figure S2**). These results suggested that different L/D cycle had significant impacts on the lettuce phenotype. Moreover, contrary to previous understanding of the L/D cycle of plants, lettuce grown in L8/D4 scenario grew taller.

### 3.2 Lettuce physiological index by L/D cycle

**Figure 4:**
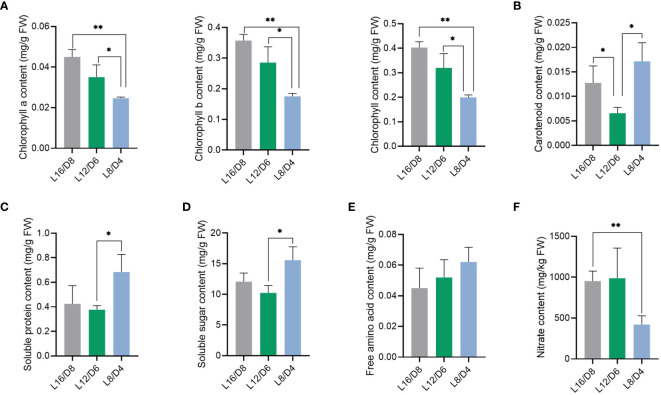
Effect of different L/D cycles on lettuce quality. The chlorophyll content **(A)**, carotenoid content **(B)**, soluble protein content **(C)**, soluble sugar content **(D)**, free amino acid content **(E)**, and nitrate content **(F)** of lettuce in three scenarios. L16/D8, lettuces experience 16 hours of light and 8 hours of darkness a cycle; L12/D6, lettuces experience 12 hours of light and 6 hours of darkness a cycle; L8/D4, lettuces experience 8 hours of light and 4 hours of darkness a cycle. The data signifies averages and the error bars indicate the standard deviations obtained from six samples that are biological replicates. *P<0.05, **P<0.01.

To investigate the impact of different L/D cycle on lettuce quality, some physiological indexes of lettuce leaves were measured. In the lettuce leaves, the chlorophyll a, b, and a + b exhibited a greater rise in the L16/D8 and L12/D6 scenarios compared to the L8/D4 scenario (**Figure 4A**) (*p*<0.05). On the contrary, the lettuce leaves exhibited a greater rise in carotenoid content under the L8/D4 scenario compared to the L16/D8 and L12/D6 scenarios (**Figure 4B**) (*p*<0.05). Carotenoid, as auxiliary pigments in plant photosynthesis, restored the growth of lettuce mediated by chlorophyll reduction in the L8/D4 scenario by compensating for the use of visible light radiation. In addition, we found that L/D cycle increased the quality of lettuce. The contents of soluble protein and soluble sugar in lettuce leaves were notably higher under the L8/D4 scenario compared to the L12/D6 scenario, indicating an increase of 3% and 5%, respectively (**Figures 4C, D**) (*p*<0.05). Moreover, it experienced a slight decrease under the L16/D8 scenario compared to the L8/D4 scenario, yet it did not attain statistical significance. There was no significant difference in total free amino acid content among the three scenarios (**Figure 4E**). Nevertheless, when comparing the nitrate levels in lettuce leaves, we found that there was a significant decrease of approximately 50% in L8/D4 scenario comparing that under L16/D8 and L12/D6 scenarios (*p*<0.05) (**Figure 4F**).

### 3.3 Transcriptome analysis of lettuce leaves

RNA-seq analysis was conducted on the lettuce leaves to investigate the underlying mechanism behind the different phenotypes observed in lettuce grown at L16/D8, L12/D6, and L8/D4. For transcriptome sequencing, a total of 9 RNA samples were sent, with each experiment comprising three biological replicates. Biological replicates displaying a strong correlation (R^2^>0.8) in gene expression levels (**Supplementary Figure S3**). The RNA-Seq data of 9 cDNA libraries (L16/D8_1, L16/D8_2, L16/D8_3, L12/D6_1, L12/D6_2, L12/D6 _3, L8/D4_1, L8/D4_2, and L8/D4_3) were summarized in **Supplementary Table S1**. Following the completion of quality control, we obtained a total of 66.94 Gb of clean data. The content of Q30 reached 95.83% throughout the 9 samples. A total of 123,569 transcripts were obtained in the filtered assembly. Most of the sequence lengths were concentrated below 20000bp (**Supplementary Figure S4**).

There were 42,796 non-redundant high-quality unigenes annotated by searching a database of common functions. From the COG, EC, GO, KEGG, and Pfam databases, a total of 40,332 (94.24%), 9097 (21.26%), 20,882 (48.79%), 20,052 (46.85%), and 39,527 (92.36%) annotated unigenes were obtained, respectively (**Supplementary Figure S5**). The PCA analysis showed an obvious differentiation between the three scenarios, suggesting that the differences among the nine sample bioreplicates aligned with the anticipated experimental design (**Supplementary Figure S6**).

### 3.4 Analysis of differential expression genes responding to different L/D cycle

**Figure 5:**
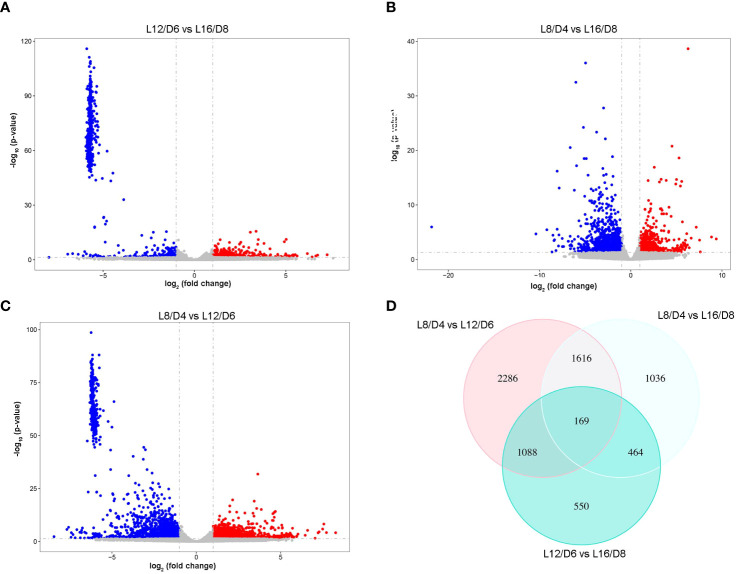
The statistical analysis of DEGs in reaction to various L/D cycle. The scatter plot displays the DEGs in L12/D6 vs L16/D8 **(A)**, L8/D4 vs L16/D8 **(B)**, L8/D4 vs L12/D6 **(C)**. Log_2_ (fold change)< −1, p- value< 0.05. Blue, down-regulated genes; red, up-regulated genes; gray, undifferentiated genes. **(D)** Venn diagram of DEGs among three scenarios. L16/D8, lettuces experience 16 hours of light and 8 hours of darkness a cycle; L12/D6, lettuces experience 12 hours of light and 6 hours of darkness a cycle; L8/D4, lettuces experience 8 hours of light and 4 hours of darkness a cycle.

The fragments per kilobase of transcript per million reads mapped (FPKM) method were used to predict gene transcriptional accumulation. By comparing the expression level under different L/D cycle (L16/D8 vs L12/D6; L16/D8 vs L8/D4; L12/D6 vs L8/D4), genes that differentially up-regulated and down-regulated were screened out (Log2FoldChange > 1, adjust padj< 0.05). The results showed that 2270 DEGs were found between the L16/D8 and L12/D6 scenarios. And there were 2131 upregulated genes and 139 downregulated genes (**Figure 5A**). Furthermore, 3284 DEGs were found between the L16/D8 and L8/D4 scenarios; among these genes, 1338 were found to be upregulated, while 1946 were downregulated (**Figure 5B**). The numbers of DEGs between L12/D6 and L8/D4 scenarios were 5159. The upregulated genes were 2523, and downregulated genes were 2636 (**Figure 5C**). In addition, venn diagram were used to summarize DEG counts between all combinations of L16/D8, L12/D6, and L8/D4 scenarios. And there were 167 common DEGs between the three comparison scenarios (**Figure 5D**)

**Figure 6:**
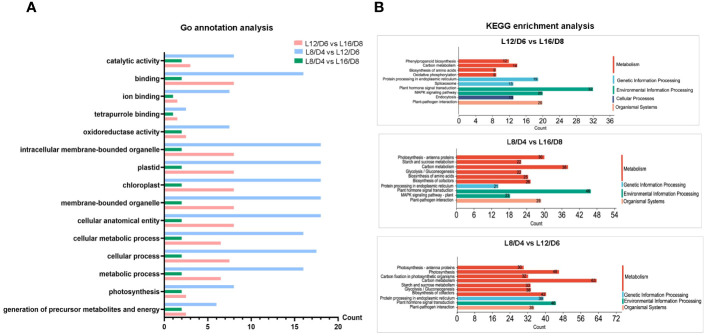
The analysis of gene function and pathway in L12/D6 vs L16/D8, L8/D4 vs L16/D8, and L8/D4 vs L12/D6. Go annotation analysis **(A)** and KEGG enrichment analysis **(B)** on the comparisons between L16/D8, L12/D6, and L8/D4. L16/D8, lettuces experience 16 hours of light and 8 hours of darkness a cycle; L12/D6, lettuces experience 12 hours of light and 6 hours of darkness a cycle; L8/D4, lettuces experience 8 hours of light and 4 hours of darkness a cycle.

By Go annotations, we discovered that the DEGs exhibited enrichment in the categories of “cellular process”, “cellular metabolic process”, and “metabolic process” within the GO classification of “biological process”. The DEGs were found to be enriched in terms such as “chloroplast”, “plastid”, and “intracellular membrane-bound organelle” within the “cellular component” categories. In the “molecular function” categories, the DEGs were enriched in “binding” and “catalytic activity” terms (**Figure 6A**). By conducting KEGG pathway analysis, we enriched the top 10 DEGs and observed their significant enrichment in various signal transduction and metabolic pathways, such as “photosynthesis”, “plant hormone signal transduction”, and “carbon metabolism” (**Figure 6B**). These results laid a solid foundation for the study of different L/D cycle adaptation mechanisms of lettuce.

### 3.5 Analysis of weighted gene co-expression network for genes associated with sensitivity to L/D cycles in lettuce

**Figure 7:**
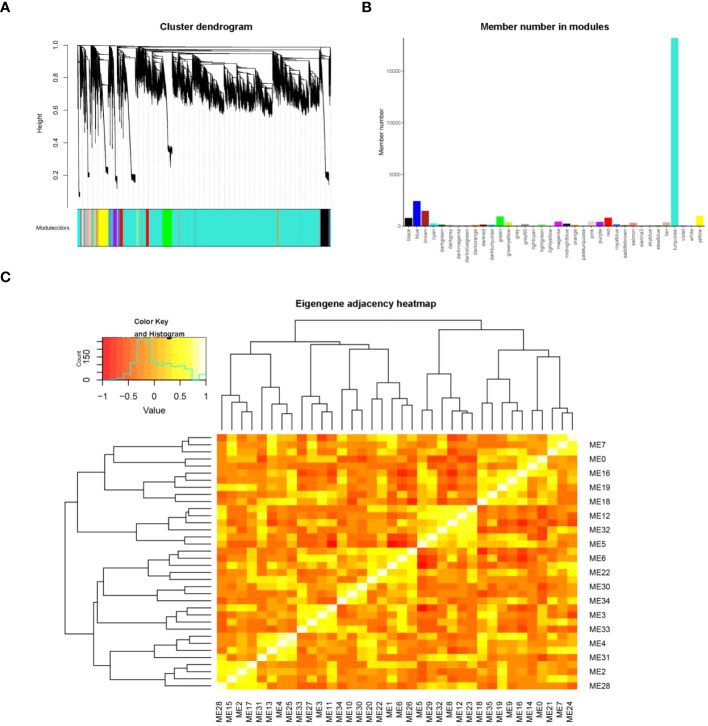
WGCNA analysis for genes related to L/D cycle sensitivity. **(A)** The cluster dendrogram of genes according to a topological overlap matrix (1-TOM). Different colors were used to display the genes divided into various modules in the dendrogram. The dendrogram was constructed using the optimal soft threshold. **(B)** The member numbers in 36 gene modules, which were ranged from 47 to 18221. **(C)** The eigengene adjacency heatmap in modules.ME represents the modules eigengene.

In order to identify the genes associated with the L/D cycle in lettuce, we performed a WGCNA analysis. When the scale-free fit index is 0.8, the minimum soft threshold for constructing scale-free networks is 9. Hence, the value of 9 can be chosen as the most suitable soft threshold for further analysis (**Supplementary Figure S7**). The co-expression network was constructed using the optimal soft threshold, dividing the genes into various modules, and conducting the cluster dendrogram(**Figure 7A**). From the results of WGCNA, 36 gene modules were identified, and marked with different colors. The member number in modules ranged from 47 to 18221 (**Figure 7B**). By constructing cluster tree and correlation heatmap, the interaction between modules was explored. These 36 modules could be aggregated into two clusters with a high degree of interactive connectivity (**Figure 7C**).

### 3.6 Module identification and functional enrichment analysis

**Figure 8:**
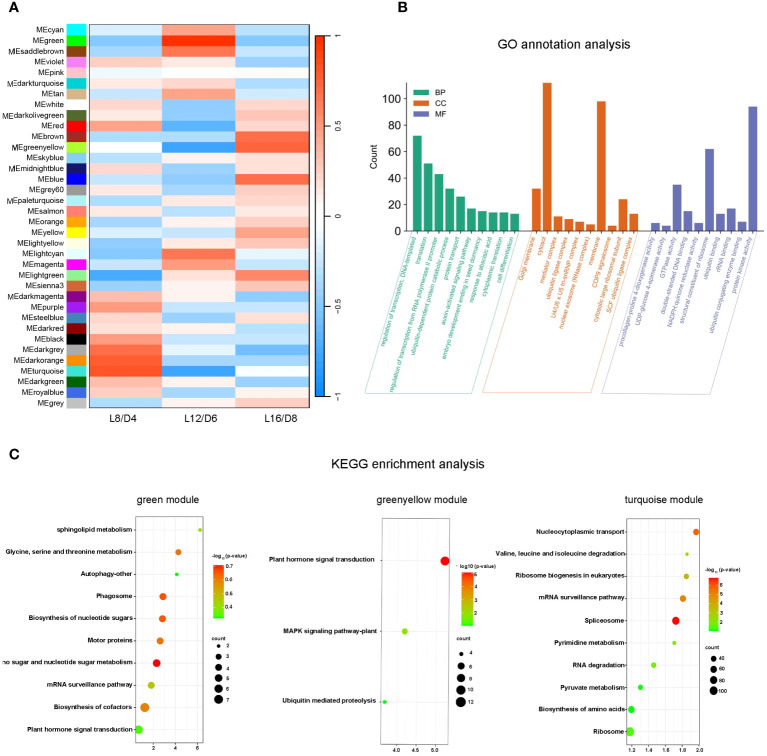
Module identification and functional enrichment analysis. **(A)** The correlation coefficients between traits and modules. ME represents the modules eigengene. L16/D8, lettuces experience 16 hours of light and 8 hours of darkness a cycle; L12/D6, lettuces experience 12 hours of light and 6 hours of darkness a cycle; L8/D4, lettuces experience 8 hours of light and 4 hours of darkness a cycle. **(B)** Go annotation analysis for differentially expressed genes in 3 modules. The genes were classified into three main categories: BP, biological process; CC, cellular component; MF, molecular function. **(C)** KEGG enrichment analysis among three modules. p- value< 0.05.

In order to identify the module that related to the treatments of L16/D8, L12/D6, and L8/D4, we conducted a correlation analysis between treatments and modules. From the results, we found that module in turquoise was positively correlated with L8/D4, and negatively correlated with L16/D8 and L12/D6. Module in green was positively correlated with L12/D6, and negatively correlated with L16/D8 and L8/D4. Furthermore, module in greenyellow was positively correlated with L16/D8, and negatively correlated with L12/D6 and L8/D4 (**Figure 8A**).

By Go annotation, we found that the DEGs of three modules in green, greenyellow, and turquoise exhibited enrichment in the categories of “regulation of transcription”, “DNA-templated” and “translation” within the GO “biological process” terms. The DEGs were found to be enriched in the terms “cytosol” and “membrane” within the “cellular component” categories. The DEGs in the GO categories of “molecular function” were found to be enriched in terms such as “ribosome structural constituent” and “protein kinase activity” (**Figure 8B**). The KEGG enrichment analysis revealed that the green module was enriched in the pathway of “sphingolipid metabolism”, the greenyellow module was enriched in the pathway of “plant hormone signal transduction”, and the turquoise module was enriched in the pathway of “nucleocytoplasmic transport”(**Figure 8C**). These three pathways directly related to plant cell growth, differentiation and signal transduction. Overall, the eigengenes of these three modules played important roles in promoting the growth and improving the quality of lettuce.

### 3.7 Screening of hub genes in the related modules

**Figure 9:**
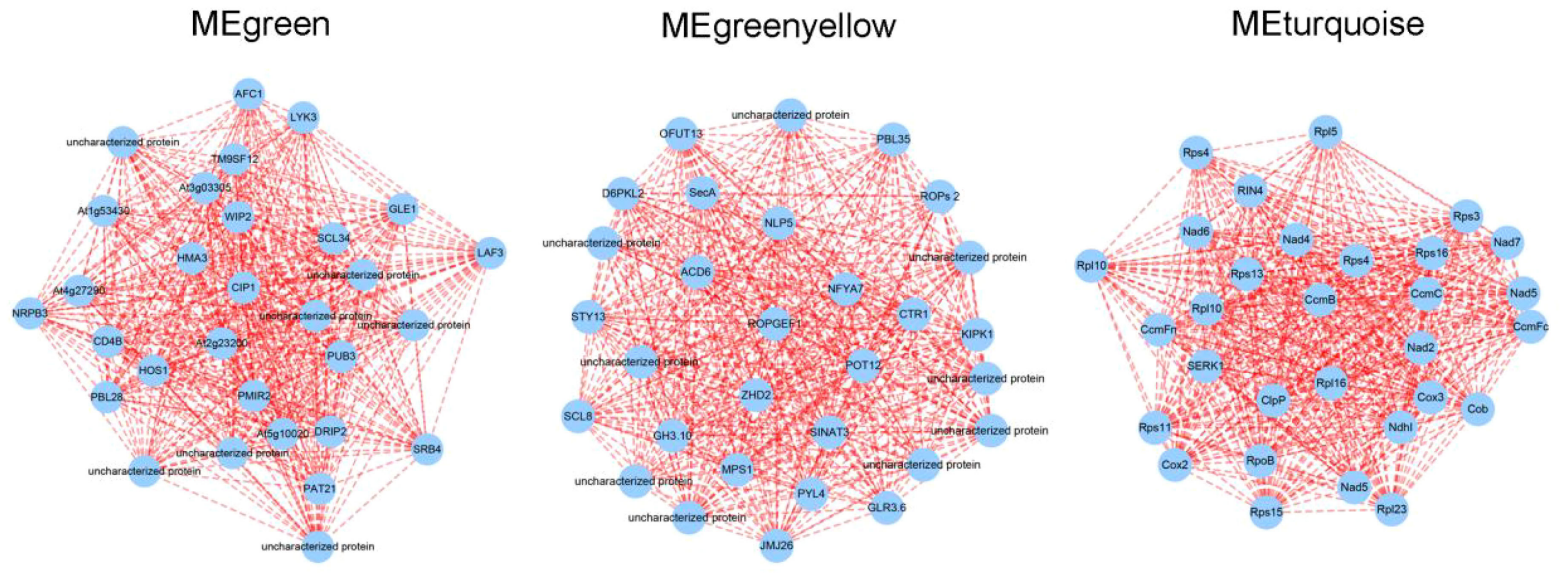
The Visual network of MEgreen, MEgreenyellow, and MEturquoise modules.

In order to explore the key gene involved in lettuce’s L/D cycle, we analyzed the top 30 nodes in the green, greenyellow, and turquoise modules with the highest connectivity through cytoscape 3.7.2 version. From the results, it was found that *CIP1* and *SCL34* were hub genes in green module. *ROPGEF1* and *ACD6* were located at the center of the greenyellow network. Furthermore, *CcmB* and *Rps4* were hub genes in turquoise module (**Figure 9**). As previously reported, *CIP1* can be ubiquitinated by *COP1*, resulting in suppression of light signal transduction and light morphogenesis (Han et al., 2020). *ROPGEF1* is associated with ABA-induced stomatal closure in plants (Li and Liu, 2012). In Arabidopsis, *CcmB* has the ability to form an ABC transporter within the mitochondria, playing a role in the maturation of cytochrome c (Rayapuram et al., 2007). Therefore, it is probable that these genes functioned as crucial switches in the alterations of plant growth and development induced by different L/D cycles.

By qRT-PCR, we examined the expression levels of *LsCIP1*, *LsSCL34*, *LsROPGEF1*, *LsACD6*, *LsCcmB*, and *LsRps4* at different time points throughout the diurnal cycle. From the results, we have found that the diurnal response patterns of the 6 hub genes in different scenarios were significant. In L12/D6 scenario, all the hub genes showed down-regulated expression after turning on the light, and the down-regulated expression trend slowed down 2 hours after the light was turned off. On the contrary, most of the hub genes (except *LsCcmB* in L16/D8) of lettuce grown in L16/D8 and L8/D4 scenarios were upregulated after the light was turned on, and the upregulation trend slowed down two hours after the light was turned off, while the expression trend of L8/D4 was more flat than that of L16/D8 (**Figure 2**). The difference of expression patterns in different scenarios indicates the difference of lettuce phenotype, and also illustrates that these six hub genes play important roles in regulating the growth and development of lettuce.

## 4 Discussion

**Figure 10:**
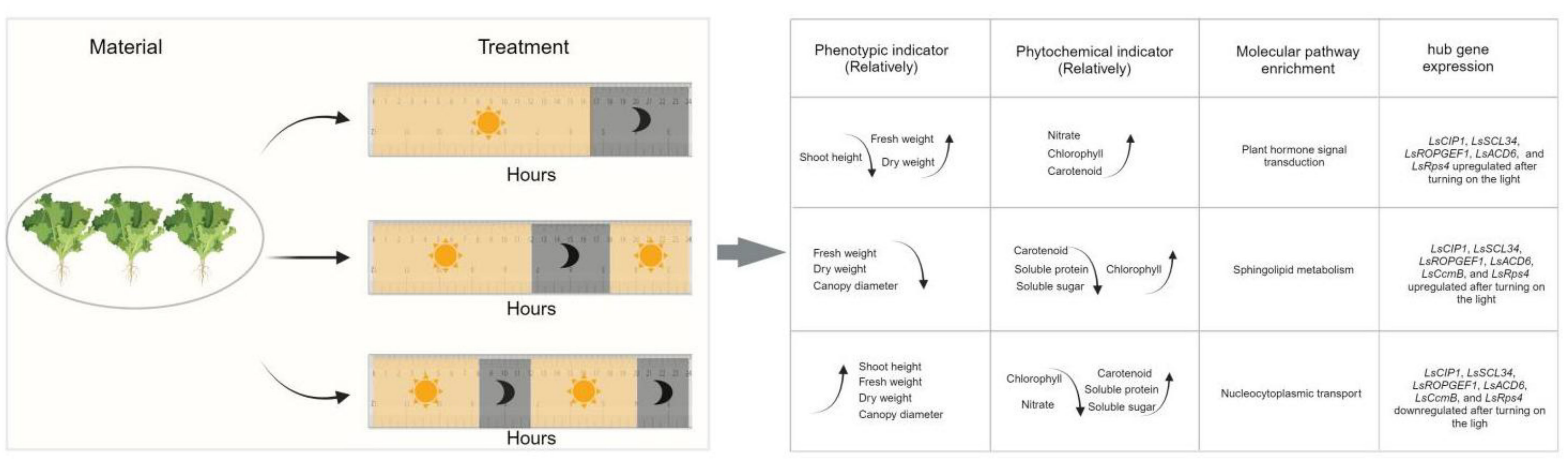
The summary diagram for the growth and morphology, phytochemical indicators, molecular pathways, and hub gene expression under all kinds of light cycles.

Circadian rhythm acts as an internal timing mechanism in plants that helps them to anticipate and align internal biological processes with these daily rhythms (Millar, 2016). In the model plants *Arabidopsis thaliana*, potato and rice crop, the circadian clock has a self-dependent mechanism, and their metabolic process is also controlled by the circadian rhythm (Kim et al., 2017; Inoue et al., 2018). Circadian clocks have two main features: endogeneity and inducibility. Endogeneity is the ability of biological physiology and behavior to operate in a certain periodic rhythm even without the external environment and other timing factors (Bell-Pedersen et al., 2005; Wijnen et al., 2006). Inducibility occurs when the constant external environment changes (like L/D cycle in this study), disrupting the endogenous mechanism of plant and forcing the biological rhythm to synchronize with the new cycle of the environment (Taiz and Zeiger, 2006). In this study, abnormal L/D cycles acted as timing factors for the plant circadian clock system. As a result of the L/D signal, plants regulate growth and physiological metabolism, and synchronize with the rhythmic change of light and dark signals (**Figure 10**).

From our study, the results showed that different L/D cycle had significant impacts on the lettuce phenotype (**Figure 10**). Lettuce experienced light cycle change twice a day (L8/D4) were significantly taller than lettuce that experienced light cycle change only once (L16/D8) (**Figure 3B**). The canopy diameter of lettuce in L8/D4 scenario was significantly larger than that of lettuce in L12/D6 scenario (**Figure 3C**). Besides, lettuce in L8/D4 scenario and L16/D8 scenario, were heavier than lettuce in L12/D6 scenario (**Figure 3D**). These results indicated that lettuce responded to light cues of different cycle by altering its morphological characteristics, and the change of L/D cycle in 24 h unit was more helpful to promote plant growth and increase yield. Moreover, based on the experimental results that lettuce can grow well under L8/D4 scenario, we propose to hypothesize that plants might not need such a long L/D cycle to develop, and the appropriate L/D phase is sufficient to complete their production/rest cycle. However, more mechanistic studies are needed to clarify this conclusion.

The morphological differences of lettuce in plant height, dry weight and canopy diameter under different L/D cycles may be partly regulated by metabolic pathways and genes. Understanding the regulatory function of L/D cycle in primary metabolism, holds immense importance in improving the yield and quality of agricultural products under controlled light environment such as greenhouse. In our study, compared with L12/D6, L16/D8 and L8/D4 treatments significantly improved the dry weight of lettuce, which may be partly due to the increased carbon assimilation capacity of lettuce. According to **Figure 3**, the soluble sugar content in L16/D8 and L8/D4 is significantly higher than L12/D6. From KEGG enrichment analysis, we also found that the top 10 DEGs were enriched in various signal transduction and metabolic pathways, such as “photosynthesis”, “plant hormone signal transduction”, and “carbon metabolism”. These results indicated that the light/dark cycle affected plants growth mainly by affecting the differential expression of genes in photosynthesis and carbon metabolism pathway. The lettuce grown under L16/D8 and L8/D4 treatment could better utilize carbon dioxide and reduce it to sugars than that under L12/D6. However, with the shortening of the L/D cycle, the pigment content of lettuce decreased (**Figure 3A**), which might be an adaptive regulation to reduce light energy capture and eliminate the potential photoinhibition caused by the shortened photocycle. The shortened L/D cycle obviously improved the utilization efficiency of pigment. In addition, according to de Barros Dantas et al. (2023), chloroplast activity is regulated by circadian genes, and the decrease in pigment content in shortened L/D cycle (especially L8/D4) could also be the result of circadian gene regulation, although its regulatory mechanism needed to be further revealed. Moreover, the difference of dry weight in L8/D4 might also be caused by the increase of photosynthetic area due to the increase of canopy diameter.

Through WGCNA analysis, we identified the core genes of *CcmB, ROPGEF1, CIP1* (**Figure 8**). *CcmB* participates in the formation of cytochrome c (Rayapuram et al., 2007). Cytochrome c is important in the process of electron transport in respiration and photosynthesis (Swierczek et al., 2010; Sun et al., 2018). Cytochrome c plays a central role in photomixotrophy in *Synechocystis* sp. by regulating the photosynthetic ability of cells (Solymosi et al., 2020). In our study, In L8/D4 scenario, *LsCcmb* was up-regulated after the light was turned on, which was opposite to the expression pattern in the other two scenarios (**Figure 9**). This indicates that lettuce in L8/D4 is participating in photosynthesis through the expression of *LsCcmb*, thus promoting growth. We hypothesized that the differential expression of *CcmB* in different treatments lead to the variation of photosynthetic ability, which affected the lettuce growth.

*CIP1* is a *COP1* interactive protein. In the process of light signal transduction, *COP1* locates downstream of the photoreceptor, playing a crucial role as the primary inhibitory factor in the transmission of light signals (Lau and Deng, 2012). *COP1* is a conserved Ring-type E3 ubiquitin ligase that related to various biological processes, including plant growth and development, mammalian cell growth, and metabolism. As an interacting protein of *COP1*, *CIP1* has a significant impact on regulating photomorphogenesis, anthocyanin synthesis and hypocotyl growth (Ren et al., 2016; Kang et al., 2021; Li et al., 2023). Taken together, we found a key gene *CIP1*, associated with lettuce growth. The expression of *CIP1* expression was activated in a 24-h-unit light cycle, thus participating in the light signaling pathway of lettuce, and on this basis, regulated the growth and development of lettuce.

*ROPGEF1* is the hub gene in greenyellow module, which mediated ABA signaling pathway. The ABA signaling pathway is closely related to the circadian clock. The circadian clock system can regulate the production and signal transduction of ABA, thus affecting the drought resistance response of plants (Baek et al., 2020; Wang et al., 2020). Plants’ responses to water deficiency vary with the light/dark cycle (Huang et al., 2019). In our study, we found that *LsROPGEF1* expression was up-regulated after the light was turned on in L16/D8 and L8/D4 scenarios, while in L12/D6 scenario, it was down-regulated (**Figure 9**). These results indicate that *LsROPGEF1* can respond to the alternation of light/dark cycle in L16/D8 and L8/D4 scenarios, so as to activate ABA signaling pathway, assist adaptive stomatal movements, establish a balance between CO_2_ uptake and water loss, and increase plant biomass.

## Data availability statement

The datasets presented in this study can be found in online repositories. The names of the repository/repositories and accession number(s) can be found in the article/**Supplementary Material**.

## Author contributions

MD: Methodology, Writing – original draft. XT: Visualization, Writing – review & editing. ZY: Writing – original draft. XC: Investigation, Writing – review & editing. YZ: Data curation, Writing – original draft. YR: Writing – review & editing. BM: Formal analysis, Writing – review & editing. DK: Data curation, Formal analysis, Investigation, Writing – review & editing.
