# Supplementary material for: Analysis of lettuce transcriptome reveals the mechanism of different light/dark cycle in promoting the growth and quality
Source: Front Plant Sci. 2024 Jul 9;15:1394434. doi: 10.3389/fpls.2024.1394434 (PMC11263018; doi:10.3389/fpls.2024.1394434)
Supplement: Supplementary file 1 [file DataSheet_1.docx]

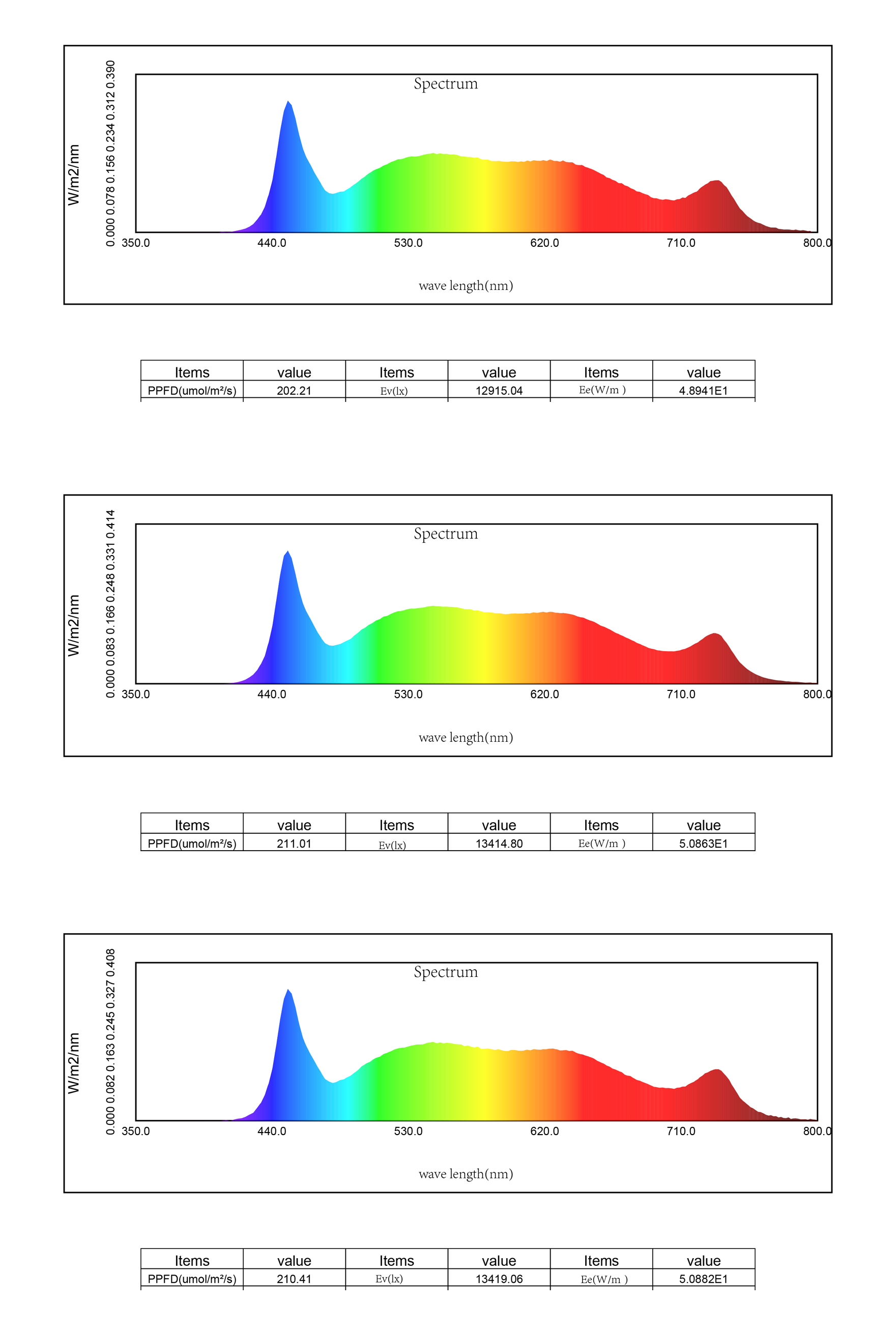


**Figure S1** The photon flux for three scenarios measured by PLA-30. The “value” in the table represents the light intensity.


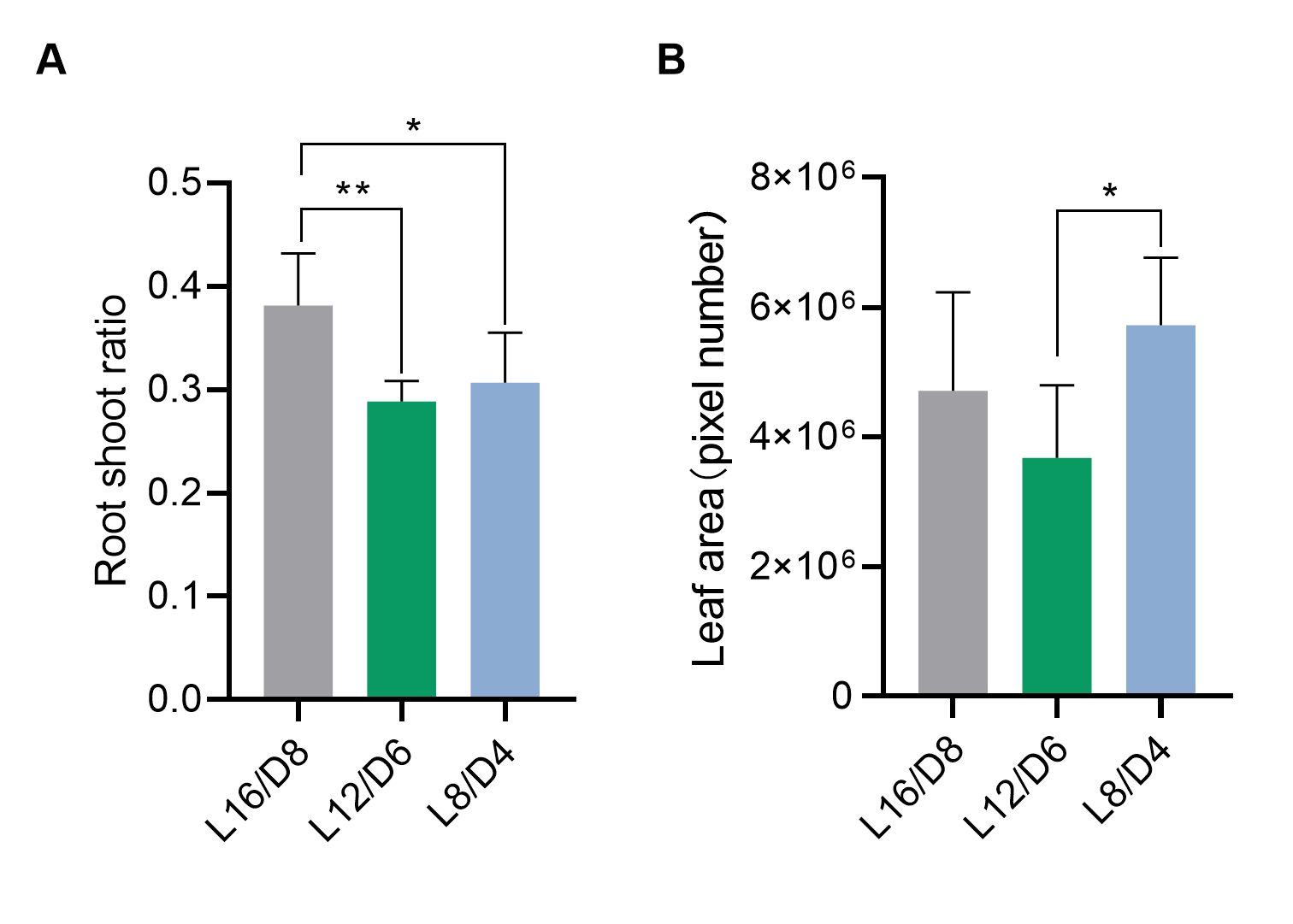


**Figure S2** The root shoot ratio (A) and leaf area (B) of lettuce growing at L16/D8, L12/D6, and L8/D4. L16/D8, lettuces experience 16 hours of light and 8 hours of darkness a cycle; L12/D6, lettuces experience 12 hours of light and 6 hours of darkness a cycle; L8/D4, lettuces experience 8 hours of light and 4 hours of darkness a cycle. The data signifies averages and the error bars indicate the standard deviations obtained from six samples that are biological replicates. **P*<0.05, ***P*<0.01.


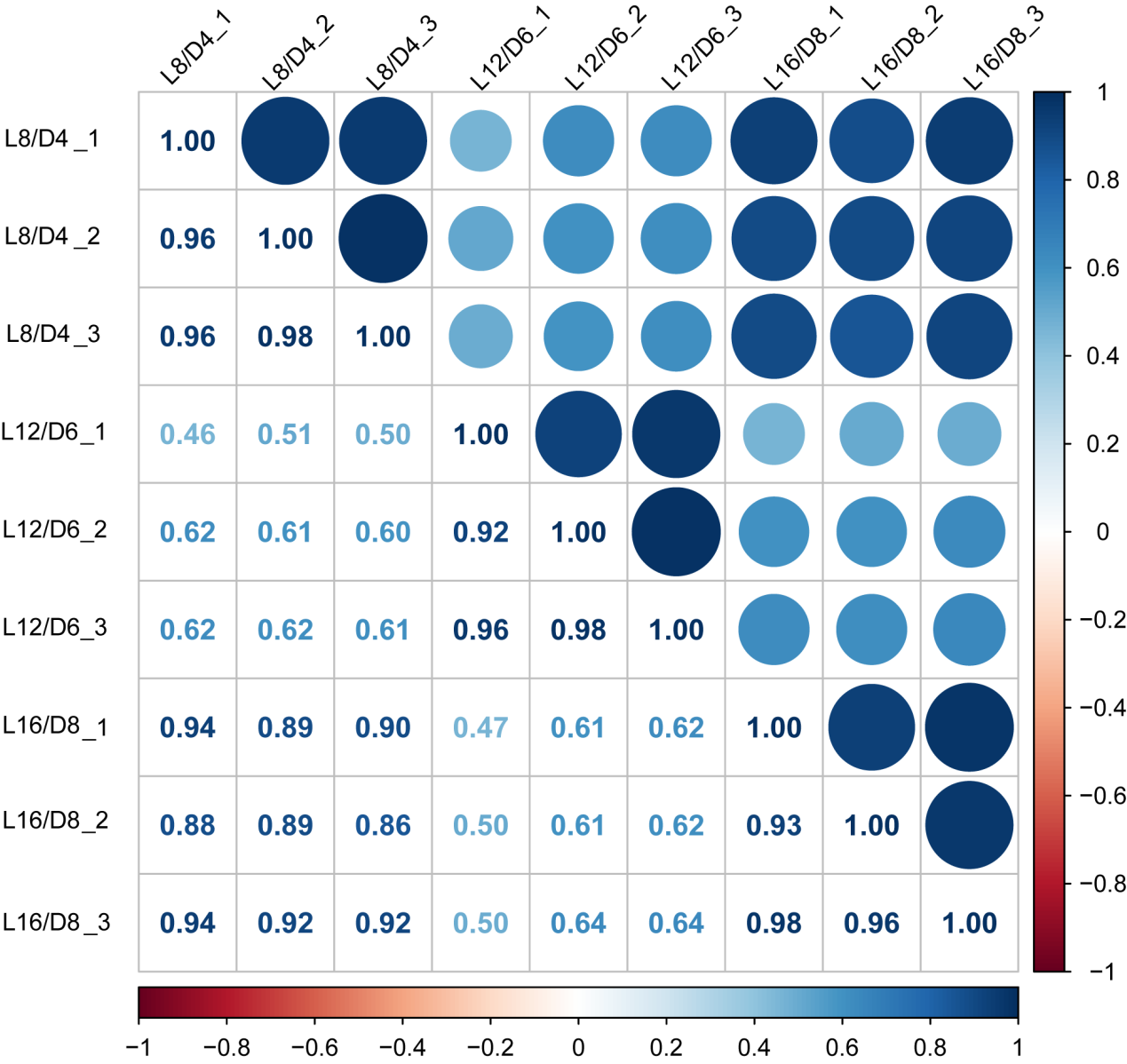


**Figure S3** The correlation coefficient analysis in all samples. The correlations of biological replicates were detected (R^2^>0.9). L16/D8, lettuces experience 16 hours of light and 8 hours of darkness a cycle; L12/D6, lettuces experience 12 hours of light and 6 hours of darkness a cycle; L8/D4, lettuces experience 8 hours of light and 4 hours of darkness a cycle.

**Table S1** The Raw data and quality control of sequencing. The correlations of biological replicates were detected (R^2^>0.8).

| **Sample ID** | **Raw reads** | **Raw bases** | **Clean reads** | **Clean bases** | **GC content(%)** | **Q30(%)** |
| --- | --- | --- | --- | --- | --- | --- |
| **L8/D4_1** | 22,357,273 | 3,375,948,223 | 21,365,020 | 3,192,908,757 | 45.35 | 96.04 |
| **L8/D4_2** | 20,514,704 | 3,097,720,304 | 19,678,967 | 2,941,192,827 | 44.81 | 96.02 |
| **L8/D4_3** | 24,642,201 | 3,720,972,351 | 23,577,255 | 3,521,700,938 | 44.59 | 96 |
| **L12/D6_1** | 22,923,962 | 3,461,518,262 | 22,020,777 | 3,290,367,549 | 48.34 | 96.09 |
| **L12/D6_2** | 21,649,731 | 3,269,109,381 | 20,650,681 | 3,083,762,597 | 49.11 | 95.97 |
| **L12/D6_3** | 21,307,965 | 3,217,502,715 | 20,406,670 | 3,045,057,075 | 48.31 | 95.91 |
| **L16/D8_1** | 22,473,817 | 3,393,546,367 | 21,410,465 | 3,197,820,082 | 45.46 | 95.99 |
| **L16/D8_2** | 20,589,806 | 3,109,060,706 | 19,721,420 | 2,945,074,731 | 46.33 | 95.83 |
| **L16/D8_3** | 20,334,304 | 3,070,479,904 | 19,324,592 | 2,886,405,309 | 45.44 | 95.95 |

**Figure S4** The distribution of transcript lengths.


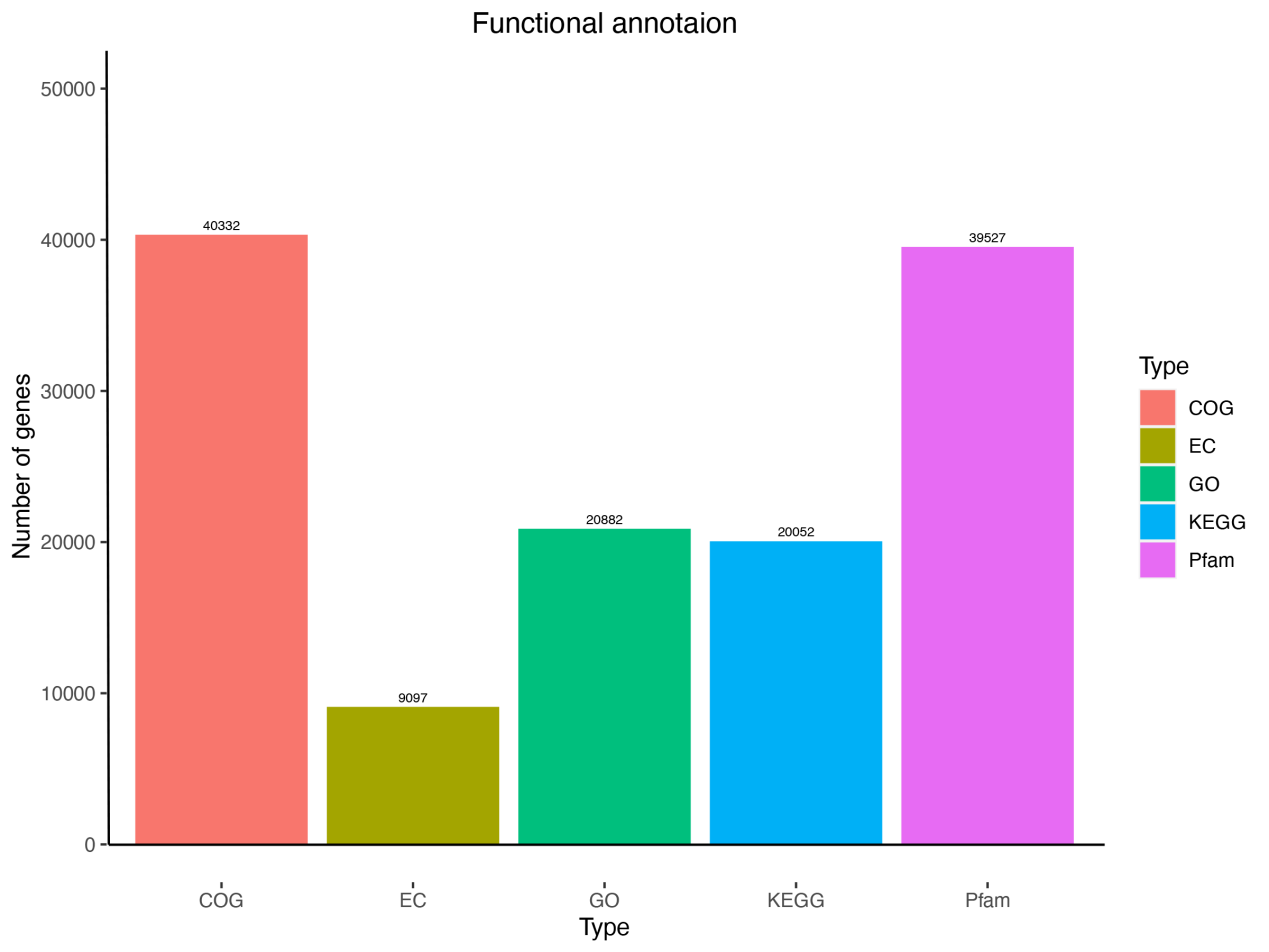


**Figure S5** The functional annotaion of the *L. sativa* L.transcriptome in COG, EC, GO, KEGG, and Pfam databases


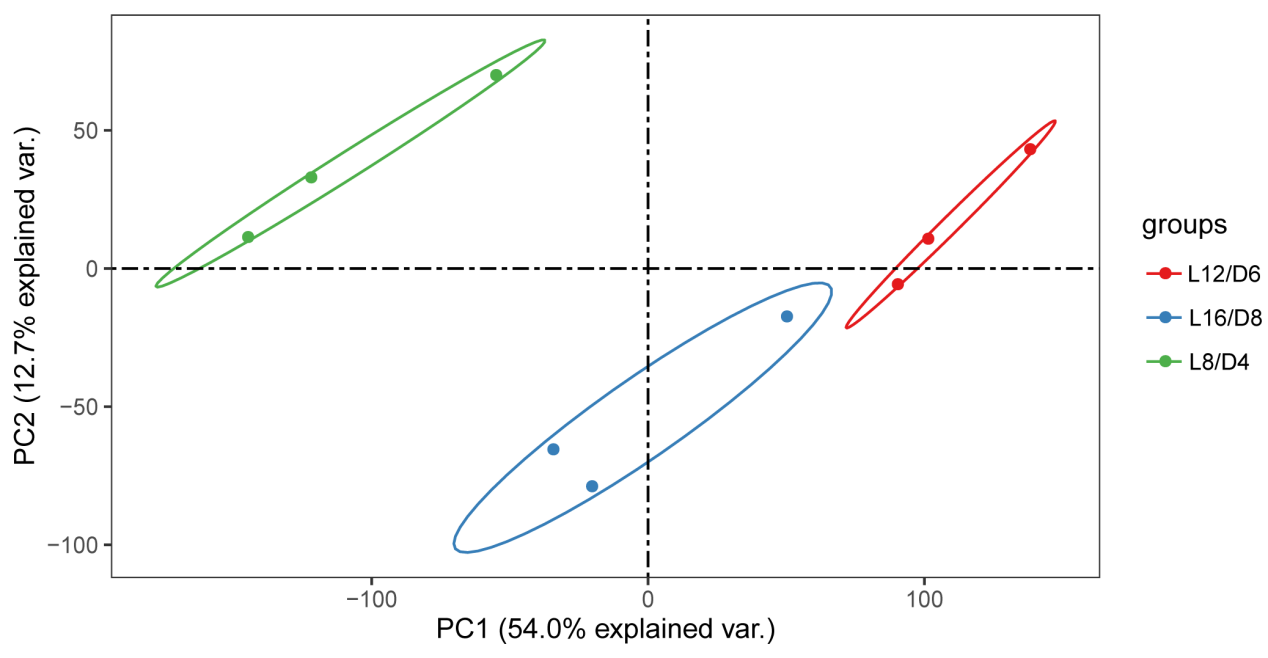


**Figure S6** Principal component analysis (PCA) of 9 samples. *p*<0.01, log_2_FoldChange>1. Red, lettuces experience 12 hours of light and 6 hours of darkness a cycle; Green, lettuces experience 8 hours of light and 4 hours of darkness a cycley; Blue, lettuces experience 16 hours of light and 8 hours of darkness a cycle


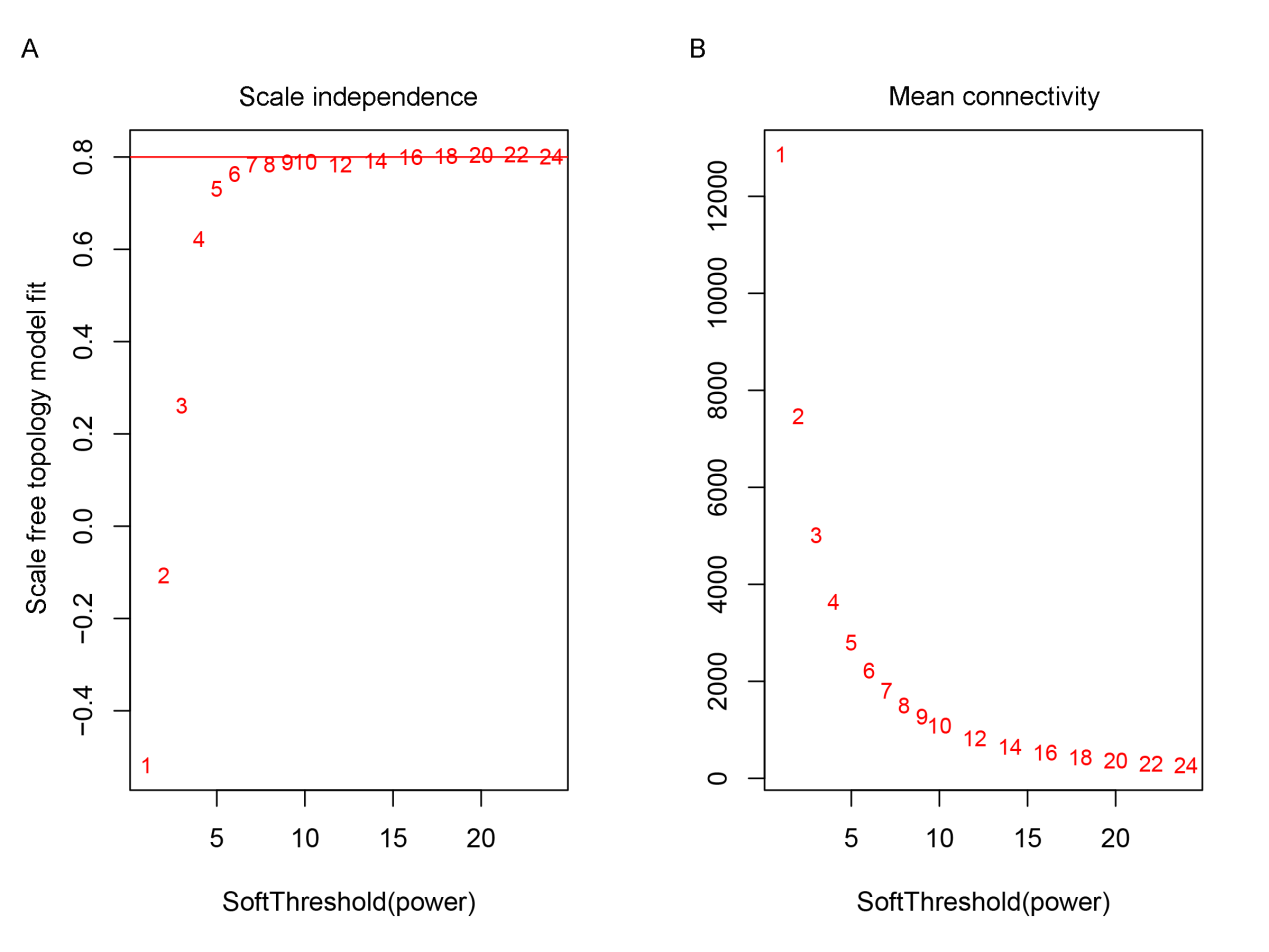


**Figure S7** The scale free topology model fit (**A**) and mean connectivity (**B**) were carried out by pick SoftThreshold function of WGCNA package, and the soft threshold 9 is calculated.

**Table S2** Primers mentioned in the article.

| **Primer name** | **Primer sequence (5'-3')** |
| --- | --- |
| qtub-F | TAGGCGTGTTAGTGAGCAGT |
| qtub-R | AACCCTCGTACTCTGCCTCTT |
| qROPGEF1-F | TTAGCGGAAAGGATGACGTG |
| qROPGEF1-R | AAACTCCTTTGCCACCTCC |
| qACD6-F | TGGAAGGGTCTGAATGGAAAG |
| qACD6-R | TGAGCCAAAACTCCGAGTATG |
| qCcmB-F | CGTGTTTTCCGTGGTTTTCC |
| qCcmB-R | TGTGATTCCAAGAGCCGAAC |
| qRps4-F | GAAAGGCAAAAGGGTCAACAG |
| qRps4-R | TTAACTAATTGGCGAGCTCCC |
| qCIP1-F | AGGATTTCGGGCATGTGATG |
| qCIP1-R | TTACCCTTTCCTCAACTTTCCC |
| qSCL34-F | TTGAAAGAGGGAATTACGAGAGG |
| qSCL34-R | CCTTCACACGCAATCACATTC |
